# GluRδ2 Expression in the Mature Cerebellum of Hotfoot Mice Promotes Parallel Fiber Synaptogenesis and Axonal Competition

**DOI:** 10.1371/journal.pone.0005243

**Published:** 2009-04-16

**Authors:** Georgia Mandolesi, Eleonora Autuori, Roberta Cesa, Federica Premoselli, Paolo Cesare, Piergiorgio Strata

**Affiliations:** 1 EBRI-Santa Lucia Foundation (IRCCS), Rome, Italy; 2 Department of Neuroscience and National Institute of Neuroscience - Italy, University of Turin, Turin, Italy; Chiba University Center for Forensic Mental Health, Japan

## Abstract

Glutamate receptor delta 2 (GluRδ2) is selectively expressed in the cerebellum, exclusively in the spines of the Purkinje cells (PCs) that are in contact with parallel fibers (PFs). Although its structure is similar to ionotropic glutamate receptors, it has no channel function and its ligand is unknown. The GluRδ2-null mice, such as knockout and hotfoot have profoundly altered cerebellar circuitry, which causes ataxia and impaired motor learning. Notably, GluRδ2 in PC-PF synapses regulates their maturation and strengthening and induces long term depression (LTD). In addition, GluRδ2 participates in the highly territorial competition between the two excitatory inputs to the PC; the climbing fiber (CF), which innervates the proximal dendritic compartment, and the PF, which is connected to spiny distal branchlets. Recently, studies have suggested that GluRδ2 acts as an adhesion molecule in PF synaptogenesis. Here, we provide *in vivo* and *in vitro* evidence that supports this hypothesis. Through lentiviral rescue in hotfoot mice, we noted a recovery of PC-PF contacts in the distal dendritic domain. In the proximal domain, we observed the formation of new spines that were innervated by PFs and a reduction in contact with the CF; ie, the pattern of innervation in the PC shifted to favor the PF input. Moreover, ectopic expression of GluRδ2 in HEK293 cells that were cocultured with granule cells or in cerebellar Golgi cells in the mature brain induced the formation of new PF contacts. Collectively, our observations show that GluRδ2 is an adhesion molecule that induces the formation of PF contacts independently of its cellular localization and promotes heterosynaptic competition in the PC proximal dendritic domain.

## Introduction

The GluRδ2 subunit is selectively expressed in the cerebellum, and at the mature stage it is targeted to the PC spines of the distal dendritic domain that is innervated by the PF input [1], [2]. Although GluRδ2 is structurally similar to ionotropic glutamate receptors, it has no channel function and its ligand is unknown. Its localization to PF-PC synapses ensures that an adequate number of PF synaptic contacts are maintained and that long-term depression (LTD)—a form of synaptic plasticity that subserves motor learning—is induced [3], [4].

PCs also receive inputs from CFs that abut clusters of spines in the proximal dendritic compartment. GluRδ2 is transiently expressed in these spines during development [2] and reappears in the mature stage after electrical activity block [5], [6], during which the cerebellar cortex is reversibly rewired. Moreover, new spines appear in the proximal dendritic domain, express GluRδ2, and are innervated by PFs. Therefore, the active CF has been proposed to repress spinogenesis in the area around its varicosities and downregulate GluRδ2 expression in its own spines. The lack of CF repression renders the postsynaptic membrane more receptive to the competitive input that invades the proximal dendritic domain. In contrast, in the GluRδ2 knockout mouse, CFs extend to the distal dendrites, thus “invading” the PF territory, where nearly half of the spines are not innervated [7]. It has been suggested that in the distal domain, GluRδ2 not only stabilizes PF synapses but also limits CF innervation to the PC proximal dendritic domain [7].

The GluRδ2-null mice, hotfoot and knockout, have free spines in the distal dendritic domain due to a loss of PF innervation [8] and a mismatch between the pre- and postsynaptic compartments [9]–[12], which indicates dysfunctional adhesion. Recently, Uemura and Mishina (2008) [13] reported that GluRδ2 expression in non-neuronal cells induces cultured cerebellar granule cells to form junctions that have synapse-like properties. By ultrastructural analysis of the same *in vitro* model, we confirmed that GluRδ2 regulates presynaptic differentiation of granule cell axons, although it is unclear whether GluRδ2 induces the formation of PF synaptic contacts *in vivo*. In particular, we do not know whether its expression in mature PCs in hotfoot mice (PC-ho) recovers PF-PC synapses.

To this end, we used a lentiviral vector-mediated rescue approach to take advantage of its ability to effect long-lasting expression of the transgene [14], [15], compared with other viral vector-based rescue approaches in studies of GluRδ2 [16]–[18]. We also cloned regulatory sequences to drive expression in specific cell types. We selected the Pcp2 (L7) promoter as a PC-specific promoter [19], [20]. Although the transgene primarily was expressed in PCs, we also observed ectopic expression in Golgi cells that were innervated by the PFs. Thus, we were able to study the effects of GluRδ2 expression in both cell types, in which GluRδ2 increased the number of PF synapses. Moreover, in the proximal dendrites of PCs that expressed GluRδ2, we observed a marked change in spine density and CF varicosity distribution. These data demonstrate that GluRδ2 is an adhesion molecule that organizes the architecture of PC innervations.

## Results

### Ultrastructural analysis of granule cell terminals of GFP- and GluRδ2-expressing HEK293 cells

**Figure 1:**
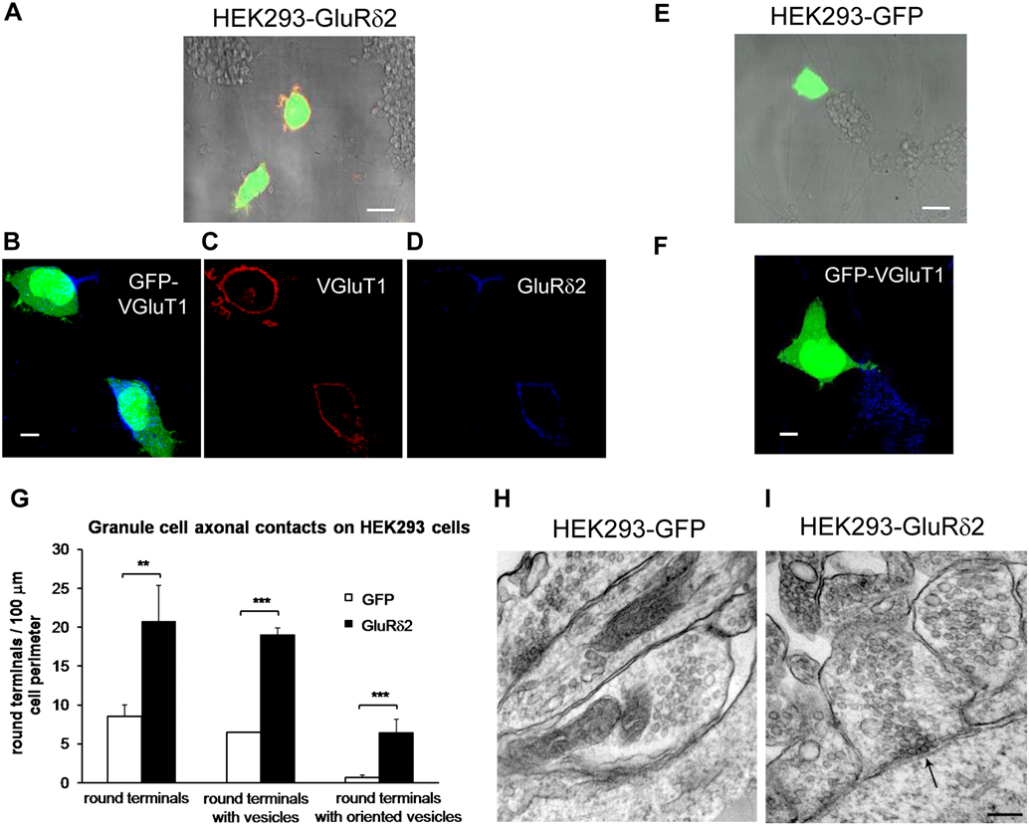
GluRδ2 expressed by HEK293 promotes formation and differentiation of GC axonal contacts. (A–F) Merge of light microphotographs of GCs in coculture with fluorescent 293 cells expressing GFP and GluRδ2 (in red) (A) or GFP alone (E). The corresponding immunofluorescence images are magnified as a single optical section in B–F. The GluRδ2 labeling around the 293 cell perimeter is shown in (C). GluRδ2 expression induces an increase in synaptic contacts, as indicated by the corresponding VGluT1 labeling (B–D). No contacts are visible around the perimeter of the 293-GFP cells; the blue labeling indicates synaptic contacts on a GC cluster (F). (G) EM quantitative analysis of the GC axonal contacts on the 293 cell perimeter. The 293-GluRδ2 cells (black columns) are in contact with a higher number of GC round terminals relative to the control (white columns); in both groups, most of the round terminals contained vesicles. In the 293-GluRδ2 cells, more terminals with vesicles oriented toward the postsynaptic membrane were observed. (H–I) EM images of differentiation of the presynaptic GC terminals induced by 293-GluRδ2 cells. (H) Contact between 293-GFP cell and a round GC terminal containing homogeneously distributed vesicles. (I) A 293-GluRδ2 cell contacted by round GC terminal containing oriented vesicles; the arrow indicates the vesicle cluster. Scale bars: A and E = 20 µm. B-C-D-F = 10 µm. H and I = 0.25 µm. *** p<0.001; ** p<0.01.

GluRδ2 expression in HEK293 cells that are cultured with cerebellar granule neurons triggers presynaptic differentiation [13]. We used a similar protocol to evaluate the ultrastructural properties of the synaptic-like contacts . Stable clones of HEK293 that expressed either GFP alone (293-GFP) or GluRδ2 and GFP (293-GluRδ2) were cultured over dissociated cerebellar granule cells (CGCs). After 1 day of coculture, we assessed the expression of GluRδ2 and vesicular glutamate transporter 1 (VGluT1), a marker of granule axon synaptic terminals [21], [22], by immunofluorescence **(** **Fig. 1A–F** **)**. We observed a significant increase in synaptic contacts on expression of GluRδ2. The mean percentage of colocalized area of VGluT1 and GFP over the entire area of GFP in each cell was 0.2 (±0.05; number of cells = 51) in GFP-transfected cells and 2.2 (±0.54; number of cells = 54) in 293-GluRδ2 cells (Student's t-test, p<0.05).

Because the effects of GluRδ2 on the morphology of such synapse-like junctions had been not investigated, we performed an ultrastructural analysis of the coculture. We first measured the density of contacts between CGC terminals and 293-GFP or 293-GluRδ2 cells. In both cultures, fibers that emerged from the GC bodies had two distinct morphological features at the junction with 293 cells—“round terminals,” which assumed the classical profile of presynaptic terminal boutons, with comparable minor and major axes lengths and the absence of cytoskeletal elements, and “elongations” that were morphologically similar to *en passant* fibers. As shown in **Fig. 1G**, the density of round terminals, expressed as the number of terminals per 100 µm of 293 cell length perimeter, was significantly higher for 293-GluRδ2 cocultures (20.7±4.7 SE) than for 293-GFP (8.5±1.4 SE) (Student's t-test, p<0.01). In addition, the mean length of presynaptic membrane that abutted 293 cells was not significantly different (0.61 µm±0.04 SE for 293-GluRδ2 cells versus 0.67 µm±0.04 SE for 293-GFP, p>0.05), suggesting that GluRδ2 increases the number of contacts but not their lengths. In contrast, the density of elongated contacts with 293 cells was similar in both experimental groups (0.17±0.04 SE in the GluRδ2-293 culture versus 0.15±0.02 SE for GFP-292, p>0.05).

Next, we analyzed the morphology of the presynaptic structures. In both experimental groups, most round terminals contained vesicles **(** **Fig. 1G** **)**. We defined two subclasses of round terminals: those that had homogenous vesicle distribution and those that had vesicles that were oriented toward 293 cell membranes. Interestingly, 293-GluRδ2 cells had significantly more presynaptic round terminals that contained oriented vesicles (0.67±0.29 SE 293-GFP versus 6.45±1.71 SE 293 GluRδ2; Student's t-test, p<0.001) **(** **Fig. 1G–I** **).** These data indicate that GluRδ2 expression in non-neuronal cells triggers the formation of contacts with GC axons and suggest that interactions with a presynaptic protein regulate vesicle clustering.

### *In vivo* injection of L7-GluRδ2/GFP virus in hotfoot mice

**Figure 2:**
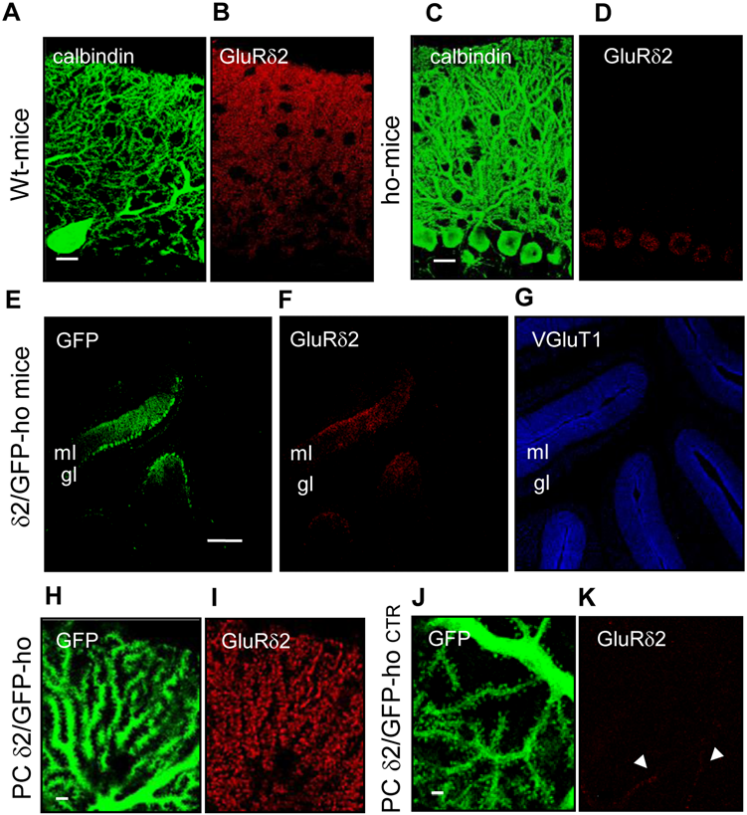
*In vivo* injection of L7-GFP and L7-GluRδ2 viruses in the mature cerebellum of ho-4J mice (δ2/GFP-ho mice). (A–D). Immunostaining of PCs labeled with anti-calbindin (green) and anti-GluRδ2 (red) antibodies in wild-type mice (A–B) and ho-4j mice (C–D). In the ho-4j mice the GluRδ2 truncated protein is retained in the PC soma. (E–F–G) Immunostaining of a cerebellar sagittal section from δ2/GFP-ho mice 4 weeks after *in vivo* injection. The infected PCs express GFP (green, E) and GluRδ2 (red, F). VGluT1 antibody (blue, G), used as an endogenous marker, labels the mossy fibers and the PF terminals in the granular (gl) and molecular layers (ml), respectively. (H–K) High magnification images of PCs expressing GFP (green) and GluRδ2 (red) in the distal dendrites of δ2/GFP-ho mice. Two populations of GFP-positive PCs are shown: PCs expressing GluRδ2 (red) (H–I) and the PCs with undetectable levels of GluRδ2 (δ2/GFP-ho CTR group) (J–K). The arrowheads indicate ectopic GluRδ2 in a different cell. Scale bars: A–D = 20 µm, E–G = 200 µm, H–N = 2 µm.

We extended our study *in vivo* to determine whether the free spines that are abundant in mature GluRδ2-null PCs become innervated on expression of GluRδ2. We used the DBA ho-4j strain of the hotfoot mouse, which carries a 170-amino acid deletion of the N-terminal region of GluRδ2 [9], [23]. Because this region is essential for GluRδ2 localization to the spine membrane, its truncated form is retained inside the PC soma [24] (**Fig. 2A–D** **)**.

DBA ho-4j mice are phenotypically similar to GluRδ2 KO mice [4], [25]. In the DBA ho-4j mouse strain, numerous clusters of naked spines are in the spiny branchlets of PCs, and a mismatch between presynaptic active zones and the postsynaptic side has been described by qualitative electron microscopy [9], [11]. Persistent multiple innervation of the CF also has been reported, although the rate of multiple innervation was lower compared with GluRδ2 KO mice [11]. Spines in hotfoot mice typically emerge from the proximal dendritic compartment of PCs, which has not been observed in GluRδ2 KO mice [11].

Here, by quantitative confocal analysis, we characterized the pattern of innervation of the two excitatory inputs that impinge on PCs in DBA ho-4j mice and DBA wild-type mice. To this end, we injected viral particles that carried *Grid*-2 and GFP cDNA into the cerebellar parenchyma of adult mice. To attain chronic expression in PCs, we used a third-generation lentiviral vector and the L7 promoter—a regulatory sequence of the Pcp-2 gene that normally is expressed only in PCs and retinal bipolar neurons [26], [27].

We produced two highly concentrated viral stocks: the ‘L7-GFP’ preparation, containing virus that expressed GFP cDNA under the L7 promoter, and the ‘L7-GFP/L7- GluRδ2’ stock, which was a mixture of 2 different viral particles—L7-GFP and those that expressed *Grid-2* cDNA under the L7 promoter. The L7-GFP particles in the latter were necessary to identify the area that was transduced by the virus after injection.

We studied two groups of adult homozygous hotfoot mice: the δ2/GFP-ho group (n = 5), injected with the L7-GFP/L7-GluRδ2 viral mix, and the GFP-ho group (n = 5), treated with L7-GFP control virus. In **Fig. 2E–G**, GFP and GluRδ2 expression were detectable in the hotfoot background of a cerebellar section 4 weeks after injection, identified by VGluT1 labeling. In these mice, we distinguished two populations of GFP-positive PCs; one that expressed the GluRδ2 subunit (**Fig. 2H–I**) and another that had undetectable levels of GluRδ2 in the distal dendritic spines (**Fig. 2J–K**). It is likely that the latter population of PCs was infected only by L7-GFP virus; this group was used as an internal control and named the δ2/GFP-ho CTR group.

We also injected DBA wild-type mice (GFP-wt, n = 3) with L7-GFP virus. The L7-GFP/L7-GluRδ2 mixture was not injected into this group, because the endogenous expression of GluRδ2 limits the identification and analysis of PCs that express L7-driven GluRδ2 protein.

### Recovery of PF-PC synaptic contacts in the distal dendritic compartment in hotfoot mice

**Figure 3:**
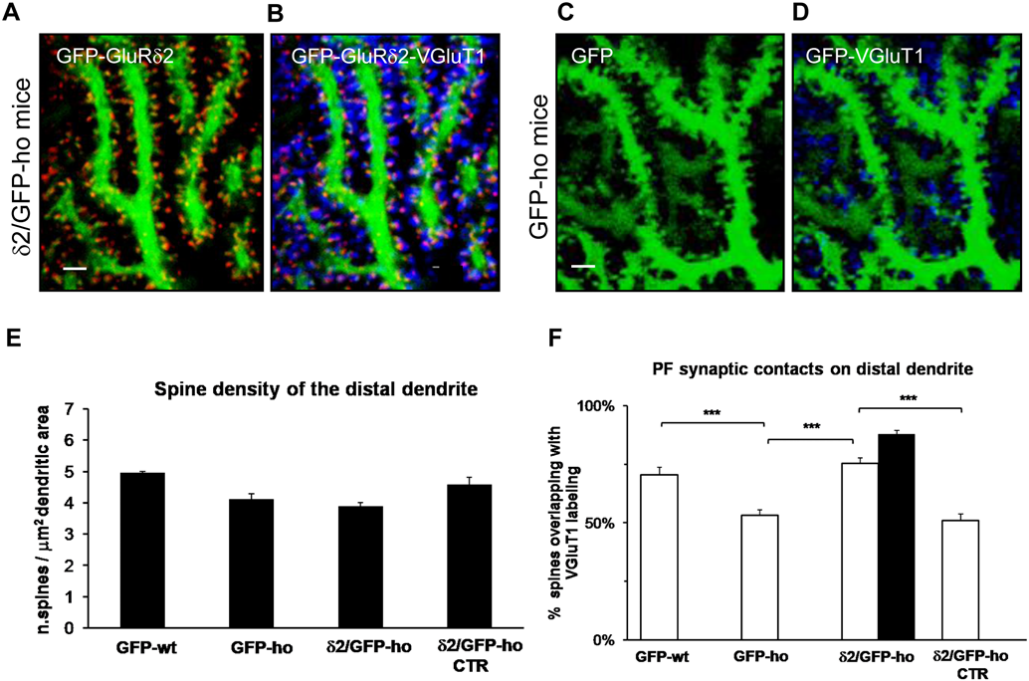
GluRδ2 promotes formation of PF contacts in the PC distal domain of δ2/GFP-ho mice. (A–D) Immunostaining of PF innervations on PC distal dendrites of δ2/GFP-ho mice (A–B) and GFP-ho mice (C–D). GFP spines bearing GluRδ2 (red, A) are contacted by PF terminals labeled by VGluT1 antibody (blue) (B). (E–F) Histograms show the mean density of spines emerging from the distal dendritic domain and the percentage of spines contacted by the PFs in this compartment. (E) The mean spine density does not change between the experimental groups (p = 0.096). (F) The mean percentage of spines overlapping with VGluT1 is increased in δ2/GFP-ho mice relative to control ho groups (GFP-ho and δ2/GFP-ho CTR), while there is no significant difference between δ2/GFP-ho mice and the GFP-wt group. In the presence of GluRδ2, indicated as the percentage of spines expressing GluRδ2 (black column), the number of PF contacts reaches that of wild-type mice. *** p<0.001. Error bars indicate SE. Scale bars:  = 2 µm.

To detect morphological changes in the cerebellar cortex, we performed immunofluorescence experiments and confocal microscopy four weeks after injection. We first observed that in δ2/GFP-ho mice, GluRδ2 was expressed in several neurons and restricted to the dendritic spines of the PC distal dendritic domain (**Fig. 3A**). Then, we investigated whether GluRδ2 induced these distal spines to establish contacts with the VGluT1-labeled PF terminals (**Fig. 3A–D**). Therefore, in each experimental group, we counted the GFP-positive spines (δ2/GFP-ho n = 1135, GFP-ho n = 1295, GFP-wt n = 925) in samples of distal dendritic segments whose diameters were less than 2 µm. In GFP-wt and GFP-ho mice, the mean spine density, expressed as the number of spines per square micrometer (µm^2^) of dendritic surface, was 4.95 (±0.062 SE) and 4.11 (±0.18 SE), respectively. In δ2/GFP-ho mice the value was 3.88 (±0.13 SE, δ2/GFP-ho) for the PCs expressing the L7-driven GluRδ2 and 4.57 (±0.25 SE, δ2/GFP-ho CTR) for the PCs only GFP-positive. The difference between the groups was not significant (one-way ANOVA, p = 0.096) (**Fig. 3E**). These results indicate that in the distal dendritic domain, the expression of GluRδ2 does not effect any increase in spine density.

Next, we determined the percentage of spines that were connected to PFs by colocalization analysis. Each GFP-positive spine was classified as positive or negative if VGluT1 colocalization was present or absent, respectively. We assumed that in the GFP-wt group, all spines in the distal compartments were connected to PF terminals. Therefore, the mean percentage value of this experimental group, which was 70.5 (±0.3 SE), was used as a control value (see methods). By analyzing the PF-linked spines in the GFP-ho group, we calculated a mean percentage of 53.2 (±0.2 SE), which was significantly different compared with the control group (one-way ANOVA, p<0.001; post hoc Holm-Sidack test, p<0.05). We also noted a significant recovery of synaptic contacts in δ2/GFP-ho mice (75.4±0.2 SE) (one-way ANOVA, p<0.001; post hoc Holm-Sidack test, p>0.05 versus control and p<0.05 versus GFP-ho), as shown in **Fig. 3F**. The same spines were analyzed for the presence of GluRδ2, wherein 88% expressed the subunit (**Fig. 3A,B,F**).

To further characterize the specific effect of GluRδ2, we analyzed GFP-positive PCs that had undetectable levels of GluRδ2 (δ2/GFP-ho mice CTR) in the spines of the distal dendritic compartment in the same δ2/GFP-ho mice. The mean percentage value of PF-connected spines in this sample (51.0±0.3 SE) was indistinguishable from the GFP-ho group (one-way ANOVA, p<0.001; post hoc Holm-Sidack test, p<0.05 versus δ2/GFP-ho and p>0.05 versus GFP-ho), as shown in **Fig. 3F**. Collectively, these results suggest that GluRδ2 expression in the distal compartment of the PC dendrite induces the complete recovery of PF contacts in the mature cerebellum.

### Spinogenesis and axonal competition in the proximal dendritic compartment

**Figure 4:**
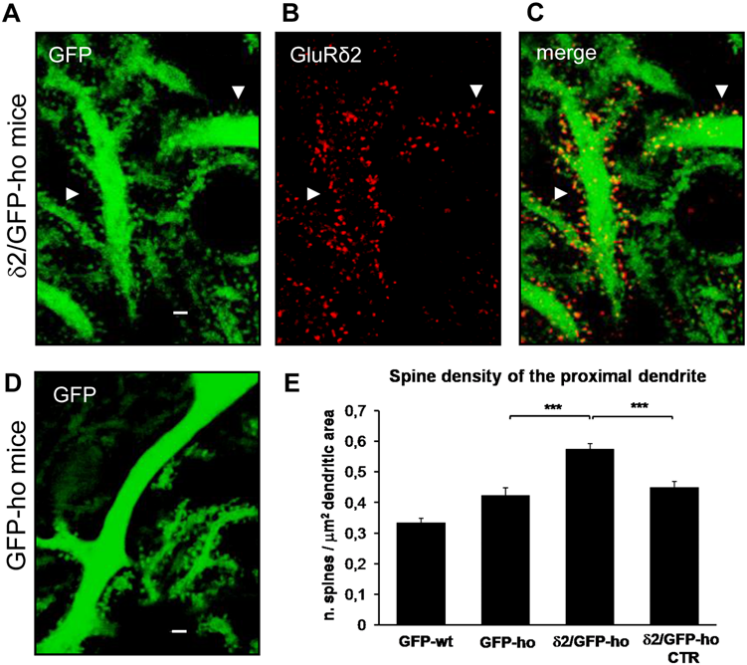
GluRδ2 induces spinogenesis in the PC proximal dendritic compartment of δ2/GFP-ho mice. (A–D) Immunostaining of PC proximal dendrites in δ2/GFP-ho (A–C) and GFP-ho mice (D). In δ2/GFP-ho mice, many new spines, expressing the GluRδ2 subunit (red) (B and C), appears in the proximal dendrite relative to GFP-ho mice (D). (E) Histogram shows the mean spine density in the proximal dendritic domain. In the presence of GluRδ2, the number of spines significantly increases relative to control groups (GFP-wt; GFP-ho and δ2/GFP-ho CTR). *** p<0.001. Error bars indicate SE. Scale bars: A–E = 2 µm.

In δ2/GFP-ho mice, we examined the proximal dendritic compartment of GluRδ2-positive PCs. Many new spines appeared in the proximal dendritic domain relative to control animals (**Fig. 4A–D**). Therefore, we measured spine density along the proximal dendrites whose diameters were greater than 2.5 µm. In the GFP-wt group, the spine density was 0.33 (±0.01 SE, n = 1434) per unit dendritic area and 0.42 in GFP-ho mice (±0.02 SE, n = 806) (**Fig. 4E**), with no significant difference (one-way ANOVA, p<0.001; post hoc Holm-Sidack test, p>0.05 versus GFP-ho). In the δ2/GFP-ho group, however, the mean density in GluRδ2-expressing PCs was 0.57 (±0.02 SE, n = 1575), 1.4-fold higher than in GFP-ho mice (one-way ANOVA, p<0.001; post hoc Holm-Sidack test, p<0.05). In the δ2/GFP-ho CTR sample, the spine density per unit area was 0.45 (±0.02 SE, n = 1198) but was not significantly different from the GFP-ho sample (one-way ANOVA, p<0.001; post hoc Holm-Sidack test, p>0.05 versus GFP-ho and p<0.05 versus δ2/GFP-ho).

In conclusion, we observed a marked increase in PC spine density in the δ2/GFP-ho group (**Fig. 4E** **)**. Interestingly, the vast majority of spines in the proximal compartment in the δ2/GFP-ho group (mean percentage 78.4±2.5 SE, n = 1198) expressed GluRδ2. Altogether, these results suggest that GluRδ2 expression in the PCs of hotfoot mice induces the formation of new spines in the proximal dendritic domain.

**Figure 5:**
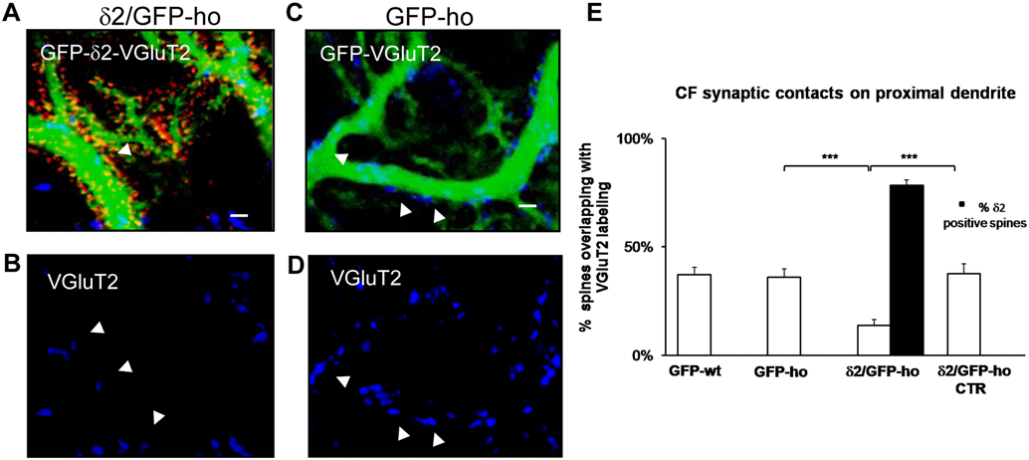
GluRδ2 causes a reduction of CF inputs on the PC proximal dendrite in δ2/GFP-ho mice. (A–D) Immunostaining of CF varicosities (blue) on the PC proximal domain of δ2/GFP-ho mice (A–B) and GFP-ho mice (C–D). (A–B) In δ2/GFP-ho mice, numerous spines bearing GluRδ2 (red, A) appear in the proximal domain. The number of CF varicosities labeled with the VGluT2 antibody (blue, A–B) is reduced relative to GFP-ho mice (C–D). The arrowheads indicate the CF varicosities in the δ2/GFP-ho that are smaller relative to the control. (E) Histogram shows the mean percentage of spines overlapping with VGluT2. A significant reduction is observed in the δ2/GFP-ho mice relative to the GFP-ho and δ2/GFP-ho CTR groups and also to GFP-wt mice. These results show that in presence of GluRδ2, indicated as the percentage of spines expressing GluRδ2 (black column), the number of CF contacts is strongly reduced. *** p<0.001. Error bars indicate SEM. Scale bars in A–D = 2 µm.

We next investigated whether the expression of GluRδ2 also affected the CF input that abuts clusters of spines in the proximal dendritic domain under normal conditions. To verify the distribution of the CF input, we immunostained VGluT2 and observed a marked decrease in innervation following induction of GluRδ2 expression (**Fig. 5A–D**). By colocalization analysis, we measured the number of VGluT2-positive spines along the dendrite that was connected to the CF terminals. In the GFP-wt control group, the mean percentage of CF-contacted spines was 37.3 (±0.3 SE, n = 1110), which was designated as the control (see methods). In GFP-ho mice, the percentage was 36.0 (±0.4 SE, n = 742) (one-way ANOVA, p<0.001; post hoc Holm-Sidack test, p>0.05) and decreased to 13.8 in the δ2/GFP-ho group (±0.3 SE, n = 1040; one-way ANOVA, p<0.001; post hoc Holm-Sidack test, p<0.05 versus all 3 groups). As shown in **Fig. 5E** **,** in PCs that did not express GluRδ2, we observed a significant difference relative to GluRδ2-positive PCs and no difference compared with the control groups (37.8±0.4 SE, n = 697) (one-way ANOVA, p<0.001; post hoc Holm-Sidack test, p>0.05 versus control and p<0.05 versus δ2/GFP-ho). These results suggest that the decrease in CF synapses in δ2/GFP-ho mice is due to a retraction of the CF input, accompanied by atrophy of the CF varicosities.

Therefore, we measured the density of CF inputs, expressed as the number of varicosities per μm^2^ of PC proximal dendritic projected area in GFP-wt (2716.00 µm^2^), GFP-ho (1258.13 µm^2^), and δ2/GFP-ho mice (1685.62 µm^2^). We observed a drastic reduction in CF density in the δ2/GFP-ho mice versus the GFP-ho and GFP-wt groups (0.12±0.03 SE, n = 582; 0.53±0.13 SE, n = 200; and 0.32±0.02 SE, n = 796 respectively) (one-way ANOVA, p<0.001; post hoc Holm-Sidack test, p<0.05 versus controls). These data show that in the presence of GluRδ2, the CF terminal changes the number of varicosities.

We also analyzed the morphology of the remaining CF input by measuring the major axis, minor axis (in micrometers), and ratio (major/minor axis length) of randomly selected varicosities in the δ2/GFP-ho, the GFP-ho, and GFP-wt groups. As shown in **Table I**, the major axis length showed a significant reduction in the presence of GluRδ2 but the minor axis did not differ. The value of the ratio (major/minor axis length) showed a significant reduction in PCs expressing GluRδ2 (**Table I**). In conclusion, the large, irregularly shaped boutons that are charateristic of GFP-ho and -wt mice become shorter and rounder on induction of GluRδ2. In previous experiments, such structural changes correlated with a reduction in the mean number of spines that were connected to each CF varicosity [28].

**Table 1:** Morphological analysis of CF varicosities in hotfoot and wild type mice

	*p*–Value	δ2/GFP-ho	GFP-ho	GFP-wt
		(n = 199)	(n = 334)	(n = 793)
**MA (**μ**m±SE)**	<0.05	* 1.11±0.04	1.24±0.03	1.31±0.02
**ma (**μ**m±SE)**	= 0.4	0.65±0.02	0.68±0.01	0.65±0.01
**MA/ma (**μ**m±SE)**	<0.001	* 1.75±0.04	1.91±0.04	2.01±0.03

Mean values of major axis length (MA), minor axis length (ma), and ratio (MA/ma); one- way ANOVA; * post-hoc Holm-Sidack test, p<0.05.

**Figure 6:**
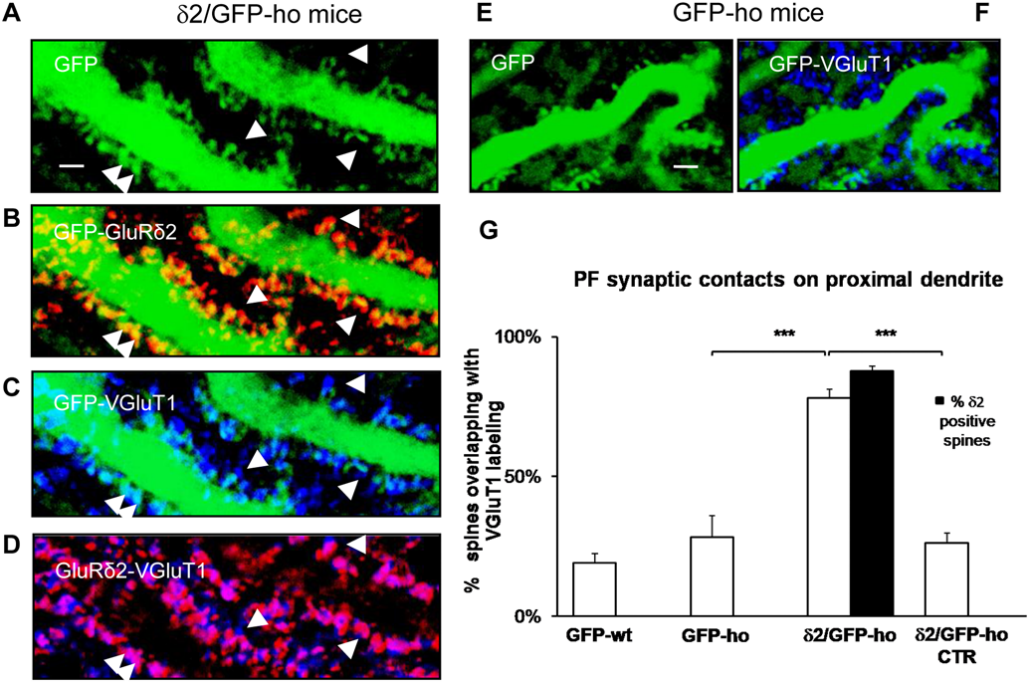
GluRδ2 promotes an increase in PF inputs on the PC proximal dendrite of δ2/GFP-ho mice. (A–F) Immunostaining of PF innervations (blue) on the PC proximal domain of δ2/GFP-ho mice (A–D) and GFP-ho mice (E–F). (A) In the δ2/GFP-ho group, numerous spines (arrowheads) bearing GluRδ2 (red, B) appear in the proximal domain, and the PF contacts, labeled with VGluT1 antibody (blue, C and D), are more numerous relative to GFP-ho mice (E–F). The overlap between GluRδ2 and the PF synaptic terminals appears as fuchsia (D). (G) Histogram shows the mean percentage of spines overlapping with VGluT1. A significant increase is observed in the δ2/GFP-ho mice relative to the GFP-ho and δ2/GFP-ho CTR groups and also to GFP-wt mice. These results show that in presence of GluRδ2, indicated as the percentage of spines expressing GluRδ2 (black column), the PF input has a competitive advantage. ***p<0.001. Error bars indicate SE. Scale bar: A–F = 2 µm.

The marked reduction in CF inputs and the presence of new spines in the proximal dendritic domain led us to examine the distribution of PF inputs (**Fig. 6A–F**). We measured the mean density of spines and counted the spines that coincided with VGluT1 expression (**Fig. 6G**). In δ2/GFP-ho mice, the mean percentage of spines that made contact with VGluT1-positive synaptic terminals increased (78.2±0.3 SE; n = 535) versus GFP-ho (28.3±0.8 SE; n = 64) and GFP-wt (19.0±0.3 SE; n = 324) groups (one-way ANOVA, p<0.001; post hoc Holm-Sidack test, p<0.05). In δ2/GFP-ho CTR mice, the mean percentage (26.2±0.03 SE; n = 501) was not significantly different from the control groups (one-way ANOVA, p<0.001; post hoc Holm-Sidack test, p>0.05) (**Fig. 6G**).

These results indicate that long-term expression of GluRδ2 in the PCs of hotfoot mice modifies the Purkinje circuitry by inducing the formation of extra spines; shrinking and reducing the number of CF varicosities; and giving a competitive advantage to the PF input. Such structural rearrangements, observed after persistent expression of GluRδ2, also suggest that under physiological conditions, the Purkinje circuitry must regulate GluRδ2 expression tightly to maintain normal architecture.

### Ectopic expression of GluRδ2 in Golgi cells of the cerebellar cortex

Next, we investigated the role of GluRδ2 in PF synaptogenesis *in vivo*. PFs innervate the PC distal dendritic compartments but also make contact with interneurons in the molecular layer of the cerebellar cortex, such as stellate and basket cells. In this layer, PFs also abut the dendritic arbor of Golgi cells. These inhibitory neurons do not express GluRδ2 but form synaptic contacts with PFs. Therefore, they represent an ideal recipient cell type to test whether GluRδ2 induces the formation of PF contacts in non-PCs.

**Figure 7:**
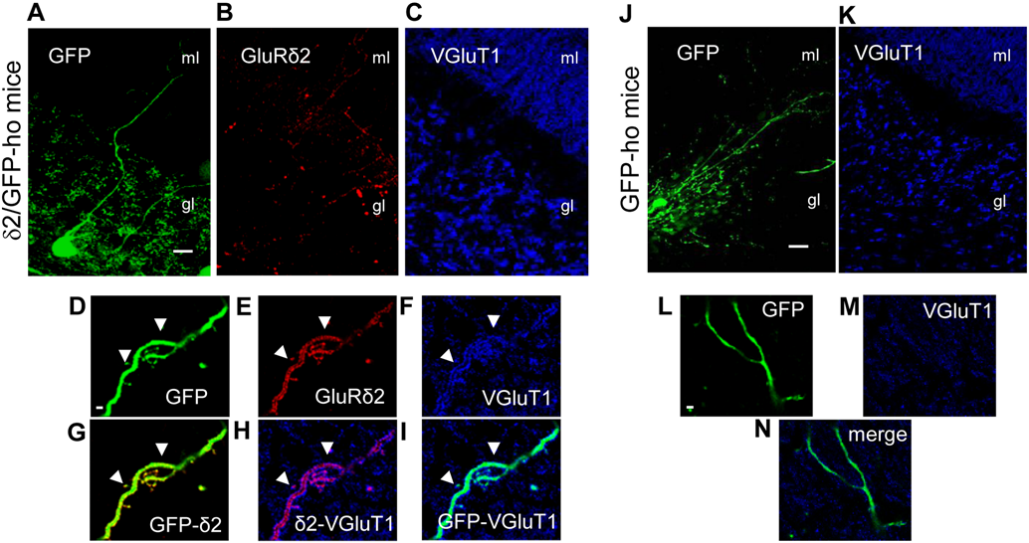
GluRδ2 increases PF contacts on Golgi cell dendrites of δ2/GFP-ho mice. (A–C and J–K) Immunostaining of transfected Golgi cells in δ2/GFP-ho mice (A–C) and in control GFP-ho mice (J–K). The cell bodies (A, J) are in the granular layer (gl), and the ascending dendrites also are visible in the molecular layer (ml). In δ2/GFP-ho mice, GluRδ2 is ectopically expressed in the Golgi dendrites (red, B). (D–I and L–N) High magnification of two Golgi cell dendrites in the molecular layer of δ2/GFP-ho and GFP-ho mice, respectively. (D–I) In a Golgi cell expressing GluRδ2 (in red, E–G–H), the dendritic area that is in contact with the PF inputs is higher (blue, F–H–I) (arrowheads) relative to that (M–N) of the Golgi cell (L) in GFP-ho mice. Scale bars: A–E = 20 µm. F–N = 2 µm.

We measured the effect of ectopic GluRδ2 in Golgi cells by driving expression of GFP and GluRδ2 with the L7 promoter (**Fig. 7**). Moreover, the hotfoot background facilitated the detection of ectopic GluRδ2 in Golgi dendrites that resided in an ‘empty’ molecular layer. Golgi cells were identified morphologically; they have a large soma below the PC layer, and the axon ramifies profusely in the granular layer to make contact with thousands of granule cells at the level of the glomeruli [29], [30]. The ascending dendrites, which branch within the molecular layer, receive inputs from the PFs either on several short neckless spines or on the dendritic shaft [29], [31], [32]. Other dendrites remain within the granule cell layer, where they make contact with mossy fibers [33], [34].

We performed immunofluorescence and confocal imaging on the 3 experimental groups, δ2/GFP-ho (n = 4; Golgi cells = 15) (**Fig. 7A–I**), GFP-ho (n = 2; Golgi cells = 7) (**Fig. 7J–N**), and GFP-wt (n = 3; Golgi cells = 18). We first analyzed GluRδ2 expression in Golgi cells dendrites and observed that GluRδ2 localized to the spines and dendritic shaft (**Fig. 7D,E,G**). Recently, it has been shown that following blockage of electrical activity, GluRδ2 is expressed not only on spines but also in excitatory postsynaptic assemblies in the smooth surface of PC proximal dendrites [35]. In our experiments, we failed to observe GluRδ2 signals in the deeper section of the granular layer. Therefore, there is no evidence that this subunit is targeted to the descending Golgi dendrites that receive mossy fibers.

**Figure 8:**
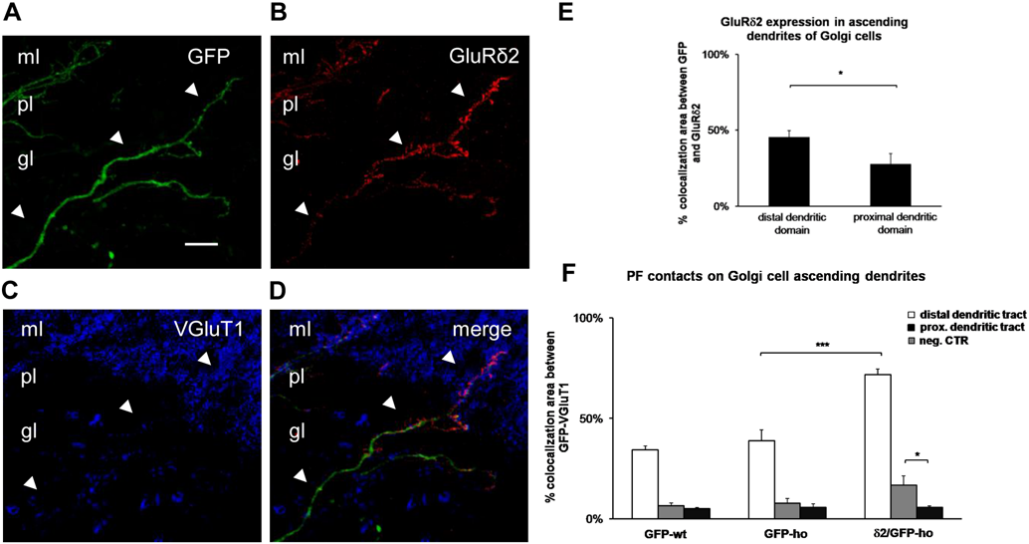
Difference in the distribution of PF contacts along the ascending domain of Golgi cell expressing GluRδ2. (A–D) Immunostaining of the ascending dendritic tract of a Golgi cell (green, A) characterized by differential localization of GluRδ2 (arrowheads) (red, B) and relative VGluT1 (blue, C) signals (D, merge). GluRδ2 expression gradually increases in the proximal domain (gl) at the level of the PC layer (pl), reaching high levels in the distal tract (ml). Although the expression of GluRδ2 is less prominent in the proximal domain, the area that is in contact with the PF inputs is significantly increased relative to both the control groups and the negative control. (E) Histogram shows the mean percentage of the GFP area that colocalizes with GluRδ2 in Golgi cell dendrites of GFP/δ2 mice. A significant reduction of GluRδ2 expression in the proximal tract is observed. (F) Histogram shows the mean percentage of the GFP area that colocalizes with VGluT1 in Golgi cells of GFP-wt, GFP-ho, and δ2/GFP-ho mice. The white columns represent the value obtained in the distal dendritic domain; the light gray columns are the value in the proximal dendritic tract; and the dark gray columns are the negative control value of colocalized GFP-VGluT1 in the rosette. The ectopic expression of GluRδ2 induces a significant increase in PF contacts in both layers. * p<0.05; ***p<0.001. Error bars indicate SE. Scale bar A–D: 10 µm.

Next, we evaluated whether GluRδ2 could extend the PF input (**Fig. 7F,H,I**) relative to the control (**Fig. 7L–N**). We measured the percentage of GFP area that was in contact with VGluT1 signal in the 3 experimental groups using colocalization software. Because GluRδ2 was differentially distributed along the ascending dendrite of the Golgi cells **(** **Fig. 8A–D** **),** we analyzed the distal dendritic region in the molecular layer, separately from the proximal tract in the granular layer.

In the distal dendritic region, GluRδ2 expression, evaluated in terms of mean percentage of GFP area that expressed the subunit (45.5±4.4 SE), increased the mean percentage of PF synaptic contacts approximately 2-fold (71.7±2.8 SE) relative to GFP-ho (38.9±5.4 SE) and GFP-wt mice (34.3±2.1 SE) (one-way ANOVA, p<0.001; post hoc Holm-Sidack test, p<0.05) (**Fig. 8E–F**).

In the proximal dendritic tract, the area of GFP that expressed GluRδ2 fell to 27.6 (±7.6 SE) (Student's t-test p<0.05). In this region, we also observed significantly fewer PF synaptic contacts (16.7±4.6) relative to the distal domain. The limited GluRδ2 expression, however, was sufficient to induce a significant increase in the PF input relative to the GFP-wt (6.6±1.2 SE) and GFP-ho (7.7±0.4 SE) groups (one-way ANOVA, p<0.05; post hoc Holm-Sidack test, p<0.05) (**Fig. 8E–F**). In contrast, no significant difference was found between the latter two experimental groups and the negative control (5.4±0.65 SE) (one-way ANOVA, p<0.001; post hoc Holm-Sidack test, p>0.05), suggesting that in the granular layer, the ascending Golgi dendrites do not receive PF inputs under normal conditions. The negative control was obtained by measuring the percentage of GFP area in the granular layer (Golgi axon) that overlapped with VGluT1 in the rosette (mossy fiber terminal).

Because the number of spines in the proximal dendritic domain of GluRδ2-expressing ho-PCs increased, we measured the spine density in ascending Golgi cell dendrites by counting the spines that emerged from both the proximal and distal dendritic tracts. Because we did not observe any difference between GFP-wt and GFP-ho mice with regard to PF inputs, we used the GFP-wt as the control.

We did not note any significant differences in the number of spines per μm of dendritic length in the GFP-wt group (0.23±0.035 SE; dendritic length = 1294.56 µm; total number of spines = 193) and in the δ2/GFP-ho mice (0.27±0.032 SE; dendritic length: 1198.61 µm; total number of spines = 238) (Student's t-test; p = 0.35). Similarly, the spine density in the proximal domain was not significantly different between the GFP-wt (0.036±0.009 SE; dendritic length = 1040.82 µm; total number of spines = 41) and δ2/GFP-ho groups (0.04±0.012 SE; dendritic length: 924.27 µm; total number of spines = 32) (Student's t-test; p = 0.74).

These results strongly suggest that GluRδ2 induces the formation of new PF contacts in non-PCs and further support a model in which GluRδ2 and PFs interact. Moreover, in Golgi cells, GluRδ2 expression does not effect an increase in the number of spines.

## Discussion

Mature cerebellar circuitry is endowed with remarkable structural plasticity, not only following damage but also under the influence of neuronal activity. Here, we provide novel evidence that such plastic events occur in mature cerebellar circuitry through changes in GluRδ2 levels in a hotfoot background. In the distal dendritic compartment of PCs, GluRδ2 promotes the recovery of PF contacts, and in the proximal dendritic compartment, spinogenesis develops and the active and intact CF terminals are displaced. In other words, the pattern of innervation in the PC shifts in favor of the PF input. Moreover, ectopic expression of GluRδ2 in cerebellar Golgi cells induces the formation of new PF contacts in the mature cerebellum. These *in vivo* observations support our *in vitro* results, demonstrating that GluRδ2 acts as an adhesion molecule.

### *In vivo* induction of PF synaptic contacts in the mature cerebellar circuitry

In GluRδ2 KO mice, GluRδ2 regulates the stabilization and strengthening of synaptic connectivity between PFs and PCs [8], [10]. This phenotype also has been observed in conditional GluRδ2 KO mice, in which GluRδ2 is downregulated by inducible and PC-specific gene targeting [12]; progressive downregulation of GluRδ2 in the adult cerebellum induces a parallel expansion of the PSD and a reduction of the presynaptic active zone, suggesting that GluRδ2 is an adhesion molecule. Consistent with these findings, Uemura and Mishina (2008) [13] observed that nonneuronal cell expression of GluRδ2 induced cerebellar granule cells in culture to form contacts that had synapse-like properties by cell adhesion assay. In particular, they demonstrated that the N-terminal domain was directly involved in stimulating these effects.

In a similar *in vitro* assay, we performed ultrastructural analysis of these contacts. We found that 293-GluRδ2 cells had more presynaptic round terminals and, most importantly, that these terminals contained vesicles that were oriented toward the target. Vesicle clustering has a crucial role in initiating synaptogenesis *in vitro* [36] and *in vivo* [37]. Therefore, our experiments support the model in which GluRδ2 expression has a morphogenic influence on presynaptic terminals and GluRδ2 acts as an adhesion molecule.

In our study, we investigated this adhesive property also *in vivo* and found that GluRδ2 alone induces PF synaptic contacts in the distal dendritic compartment in the mature cerebellum of PC-ho mice. Moreover, we observed that even in the non-PCs that normally abut PFs—Golgi cells—ectopic GluRδ2 promotes the formation of new PF contacts. In particular, the number of PF contacts increased in relation to GluRδ2 expression only in ascending dendrites, thus excluding the targeting of this subunit to Golgi dendrites that receive mossy fibers. In addition, in the molecular (distal region) and granular layers (proximal region), the ascending dendritic segment might have distinct molecular compositions and functional properties, implying that localized “polarity” exists. Under normal conditions, Palay and Chan Palay (1974) [29] reported a Golgi dendrite in the granular layer receiving synapses from axons that possibly belong to granule cells. However, no further evidence have been reported supporting this assumption. Therefore, the proximal tract of the ascending dendrite unlikely forms synaptic contacts with the granule cell axons, despite its localization in the granular layer, while the distal tract is extensively bordered by PFs. This difference can be due to several reasons [38]. One possibility is that the ability of dendrites to receive and integrate synaptic inputs requires that specific proteins, including neurotransmitter receptors, adhesion molecules, ion channels, and certain transporters, are properly localized with high spatial precision. For example, somatodendritically targeted K^+^ channels are restricted to the most proximal segments by specific targeting motifs [39]. In contrast, electrophysiological experiments have demonstrated that the number of AMPA-type glutamate receptors at distal dendrites of CA1 pyramidal neurons in the hippocampus progressively increases, as does synaptic conductance [40], suggesting that dendritic polarization occurs in the proximal-distal dimension. Accordingly, the GluRδ2 ectopically expressed in Golgi cells responds to the signals that mediate such precise targeting, resulting in polarized localization not only in the ascending and descending tracts but also in the proximal-distal dimension of the ascending tract. Moreover, following its targeting to the postsynaptic membrane, GluRδ2 may need PF synaptic contacts to maintain its localization [38], [41], [42].

In conclusion, this study makes two novel observations—GluRδ2 promotes the formation of PF synaptic contacts in adult PC-ho mice, and this effect is linked not only to its expression in PCs. The latter observation is supported by our *in vitro* and *in vivo* results. In the cocolture model, nonneuronal cell expression of GluRδ2 induces granule cell neurites to differentiate into synaptic-like structures, and in the mature cerebellum, GluRδ2 expression in Golgi cell dendrites increases PF inputs.

### Spinogenesis and heterologous axonal competition

PFs and CFs compete for PC innervation, and under normal conditions each input is confined to the distal and proximal dendritic domains, respectively, where each of them maintains its unique complement of spines. Spinogenesis is initiated in the proximal dendrites when the CF input is deleted [43]–[45] or when electrical activity is blocked by TTX [5], [46]. A similar process occurs by blockage of AMPA/kainate receptors [28]. In these cases, the new spines are innervated by the PF input, while CFs lose synaptic contact with the PCs. When the inhibition is lifted [6], [46] or when the CF-denervated PCs are reinnervated by collateral sprouting of surviving CFs [44], [45], the ectopic spines and their PFs regress fully [6]. These observations have led to the conclusion that CFs need to be active to maintain their own dendritic territory and displace competitor afferents.

GluRδ2 appears to regulate heterosynaptic competition by reinforcing PF-PC synapses. In fact, mice that lack GluRδ2 at birth [7] or following conditional deletion in the adult cerebellum [12] experience an extension of the CF input in the distal domain, where formation or strengthening of PF-PC synapses is impaired. The same phenotype exists in precerebellin-null mice. Precerebellin is a granule cell-derived secretory factor that has been proposed to regulate PF-PC synaptic formation and heterosynaptic competition in cooperation with GluRδ2 [47]. Therefore, in the distal domain, the presence of PF synapses normally limits the CF territory to PC proximal dendrites.

In contrast, in TTX-treated adult rat cerebellum, GluRδ2 is expressed in the proximal dendritic domain, where PFs form new synapses [5], [35] and the number of CF synaptic contacts with the PC decreases. We propose that to maintain its territory in the proximal compartment, CF must inhibit not only intrinsic spinogenesis but also GluRδ2 expression. The molecular mechanisms that underlie this inhibition remain unknown.

Here, we demonstrated that the induced expression of GluRδ2 in PC-ho mice, by escaping local CF control, tilts the balance of the distribution of the two excitatory inputs into PC dendrites. In particular, it displaces the active and intact CF input, favoring the PF input that extends into the hyperspiny proximal dendritic domain.

### Possible mechanisms of GluRδ2 action

With regard to the mechanisms by which GluRδ2 induces the effects described here, there are several possibilities. One mechanism proposes that GluRδ2 directly induces spine formation. Recently, AMPA and NMDA subunits have been reported to regulate spine density and size [48]–[50]. In particular, the overexpression of GluR2 induces the development and growth of dendritic spines in cultured hippocampal neurons [48], [49]. Thus, this hypothesis is unlikely because in the number of spines does not change GluRδ2-null mice. Finally, we did not observe an increase of spine density in either PC distal dendrites or Golgi cells in ho-GluRδ2 mice.

A second possibility is that GluRδ2 interferes with the molecular mechanisms that regulate activity-dependent spine-pruning that is exerted by the CF at the proximal dendrites through ionotropic AMPA/kainate receptors [28]. Excess GluRδ2 may shift the generation of tetramer AMPA receptors toward the formation of nonfunctional GluRδ2–AMPA heteromeric channels. This finding is consistent with the observation that GluRδ2, when it assembles in heterologous cells with GluR1 or the kainate receptor GluR6, forms a nonfunctional channel [51]. *In vivo* coimmunoprecipitation experiments demonstrate that endogenous GluRδ2 exists primarily as a homomeric receptor and that at least a portion is closely associated with AMPA or kainate receptors. Similarly, immunogold electron microscopy has revealed that GluRδ2 colocalizes with GluR2/3 in PC spines [1]. In our experiments, GluRδ2, by inhibiting the glutamate-induced currents of heteromeric channels [51], may mimic blockage of AMPA receptors [28]. As a consequence, the attenuation of CF synapses weakens the repression that they normally exert on the competitor afferent, leading to the emergence of new spines that bear GluRδ2 and the formation of PF synaptic contacts.

Alternatively, GluRδ2 may occupy extrasynaptic regions of CF-PC synapses or the dendritic shaft [35]. Because we demonstrated that GluRδ2 alone promotes the formation of PF synaptic contacts and PF presynaptic differentiation, we suggest that it generates PF-PC synapses in these compartments. Moreover, GluRδ2 recruits AMPA receptors to the region that faces the active zone by effecting the proper organization of pre- and postsynaptic compartments [12]. Therefore, in the presence of ectopic PF-PC synapses, competition with the CF inputs is elevated. The PF synapses progressively restrict the surrounding CF territory, and as a consequence, the lateral inhibition that is exerted by the CFs is reduced, intrinsic spinogenesis develops, and new spines that express GluRδ2 result in contact by the PFs.

Regardless of the precise mechanisms by which GluRδ2 exerts its effects, these results suggest that GluRδ2 is an adhesion molecule that induces the formation of PF contacts both *in vitro* and *in vivo* independently of its cellular localization. Moreover, GluRδ2 has the potential of inducing plastic events in cerebellar circuitry by promoting heterosynaptic competition in the PC proximal dendritic domain. For this reason, the cerebellar cortex—in particular the PCs with the PF and CF inputs—tightly regulates GluRδ2 expression and localization to maintain normal architecture under physiological conditions. If its expression is not properly controlled, GluRδ2 effects the formation of excess PF contacts, which is detrimental to cerebellar circuitry.

## Materials and Methods

### DNA constructs

cDNA that encoded mouse GluRδ2, kindly provided by Prof. N. Heintz, was first cloned into the p207.pRRLsinPPTs.hCMV.GFP.WPRE plasmid (p207) by replacing the GFP sequence, which was under control of the CMV promoter. We validated this construct in 293T cells by immunocytochemistry and Western blot. The CMV promoter was then removed to insert an L7 minigene, comprising 1 kb of the L7 promoter, 2 exons, and 1 intron and derived from the pL7-ΔAUG plasmid (a gift of Dr J. Oberdick; [52]). We cloned GluRδ2 or GFP cDNA into the second exon of the L7 gene, such that the only translational start site (ATG) was introduced by the transgene.

### Lentiviral vector production

The VSV-G-pseudotyped lentiviral vectors were generated by calcium phosphate transfection of HEK293T cells with a mixture of the 4 plasmids that are essential to produce third-generation lentiviruses (kindly provided by Prof. L. Naldini). Cells were cultured in Dulbecco's modified Eagle's medium (DMEM) supplemented with 10% fetal bovine serum (FBS), 100 U/ml penicillin G, and 100 µg/ml streptomycin at 37°C in 5% CO_2_. Cells were plated at 2.7−3×10^6^ cells in a 10-cm dish 24 h before transfection. Fifteen hours after incubation with the transfection mix at 37°C, the cells were washed with HBSS, and complete DMEM was added. Virus-containing medium was harvested 40 h after transfection, filtered through a 0.45-µm Durapore Stericup unit, and concentrated by 2 ultracentrifugation steps. The viral pellet was finally suspended in PBS with 1% BSA and stored at −80°C until use. Viral content was measured by p24 antigen enzyme-linked immunosorbent assay (RETROtek, ZeptoMetrix).

### Animals

Animals were housed according to the European Community Council Directive (86/609/CEE). The experimental protocols were designed in accordance with Italian law D.L. 116/92 and presented to the Italian Minister of Health. Adult DBA wild-type and DBA Grid2<ho4J/J>mutants (12–16 weeks; Charles River, USA) were used for the *in vivo* injection.

### *In vivo* cerebellar injection

All surgical procedures were performed under general anesthesia by avertin (100 µL/10 g), intraperitoneally injected. The animals were placed in a stereotactic frame, and microsurgery was performed to expose the upper cerebellar vermis (lobules 6–7). The particle titer of each concentrated virus was adjusted to 110,000 ng p24 per ml, and 2 µl was injected by a glass capillary (Sutter Instruments) connected to a picospritzer (Parker Inst, USA). We injected along a single track but at 4 different depths from the pial surface at a rate of 100 nl/min.

### CGC/HEK293T cell coculture

We followed the protocol described by Fu et al. (2003) [53]. Briefly, rat cerebellar granule cells (CGCs) were cocultured with HEK293T clones that stably expressed GFP or GluRδ2. Primary cultures of rat CGCs were prepared from postnatal day 5–7 (P5–7) rats. The cerebella were dissociated using a papain-based dissociation kit (Worthington Biochemical Corporation). Dispersed cells were plated at a density of 60×10^4^ cells/12-mm coverslip (Zeus super) for confocal imaging or aclar for electron microscopy (Aclar embedding film; Electron Microscopy Sciences, PA), precoated with poly-L-lysine (10 µg/ml).

The cells were cultured in basal Eagle's medium supplemented with 2 mM glutamine, 100 µg/ml gentamicin (all from Gibco, Invitrogen), and 10% bovine calf serum (HyClone) and maintained at 37°C in 5% CO_2_. The final concentration of KCl in the culture medium was adjusted to 25 mM (high K^+^). At DIV5, the medium was replaced with the low (5 mM) potassium MEM supplemented with 5 mg/ml glucose, 0.1 mg/ml transferrin, 0.025 mg/ml insulin, 2 mM glutamine, 20 µg/ml gentamicin, and cytosine-β-D arabinofuranoside 10 µM. 293-GFP and 293-GluRδ2 clones were grown in MEM supplemented with 10% fetal bovine serum, 100 U/ml penicillin, and 100 U/ml streptomycin in a 5% CO_2_ incubator. When the CGCs were at the sixth day in culture, the 293 clones were detached with trypsin and plated on CGCs at a density of 1×10^4^ cells/12-mm coverslip/aclar.

### Immunohistochemistry

Four weeks after viral injection, mice were deeply anesthetized (avertin), perfused through the aorta with ice-cold 4% paraformaldehyde, and equilibrated with 30% sucrose overnight. Thirty micrometer-thick sagittal sections were preincubated with 10% normal donkey serum solution (NDS) for 1 h at room temperature and incubated with the following primary antibodies at +4°C: monoclonal anti-calbindin 1∶2000 (D28K Swant) for 1 day, goat polyclonal anti-GluRδ2 1∶1000 (sc-26118, Santa Cruz Biotechnology, Inc) for 3 days; rabbit polyclonal antiVGluT1, and anti VGluT2 (c.n. 135302 and 35403, Synaptic Systems GmbH, Germany) 1∶500 for 1 day. After being washed with PBS, the sections were incubated with Cy-3-conjugated donkey anti-goat and Cy-5-conjugated donkey anti-rabbit secondary antibodies (Jackson ImmunoResearch) 1∶200 for 2 h at RT and rinsed in PBS 1×. Sections were mounted on poly-L-lysine-coated slides, air-dried, and coverslipped.

### Immunocytochemistry

After 24 h of CGC/HEK293T coculturing, cells on glass coverslips were fixed in 4% paraformaldehyde and 4% sucrose in 0.12 M phosphate buffer (PB) for 10 min at RT, and immunostaining was performed as described by incubating them with primary antibodies (2 h) and secondary antibodies (1 h).

### Confocal imaging

We performed double and triple immunostaining of 293T cocultures with CGCs (number of cocultures = 4) to obtain immunofluorescent and light images with an LSM5 Zeiss confocal laser-scanning microscope (Zeiss, Germany) using a 63× oil immersion lens (1.4 numerical aperture) and an additional digital zoom factor of 1.5×. We collected several optical section images (1024×1024) in the z-dimension (z-spacing, 1 µm), ensuring that each 293 cell, spanning multiple confocal planes, was fully captured.

The same confocal laser-scanning microscope was used to obtain images from double- triple-immunostained cerebellar sections. To clearly resolve the dendritic spines of PCs and the relative synaptic inputs, we used the 63× oil objective with a zoom factor of 2×. Section images (2040×2048) in the z-dimension (z-spacing, 0.5 µm) were collected, ensuring that segments of both dendrites, spanning multiple confocal planes, were fully captured.

The same immunostained cerebellar sections were acquired with a CLSM (Leica SP5, Germany) confocal laser-scanning microscope to obtain images of dendritic Golgi cells (63× oil-objective, 1.4 NA; electronic zoom factor 2.5×). Several optical section images (2040×2048) in the z-dimension (z-spacing, 0.5 µm) were captured, ensuring that segments of the dendritic tract, spanning multiple confocal planes, were fully captured.

We could not perform a blind acquisition because GFP expression provides only an estimation of the injected area, not coinfection of the L7-GluRδ2 virus. Therefore, the identification of PCs that express L7-GluRδ2 was obtained only by immunofluorescence of the protein.

### Quantitative confocal analysis

#### Purkinje cell analysis

For each cerebellum, we randomly acquired 10 to 20 images of the molecular layer. Proximal and distal dendrites were discerned according to caliber. The sizes of the distal branches reached a maximum of 2 µm, and those of the proximal branches had a larger diameter [29], [46]. We analyzed distal segments (total number = 192) that had a diameter between 0.76 and 1.9 µm and proximal segments (total number = 456) whose diameters were between 2.5 and 5.2 µm.

The explored dendritic area was calculated multiplying the mean value of the dendritic diameter by the explored dendritic length and π. The spine density evaluation was calculated by collecting the spines that emerged from 1 side of the lateral dendritic surface. Each identified spine in a given section was followed until it disappeared downstream and upstream in the image series to exclude the sample spines that emerged from other dendritic segments. To evaluate the spines that were in contact with the PF or CF synaptic terminals, we used the same images series (cross talk-free images). We used the colocalization software LSM5 (Zeiss, Germany) to identify overlapping GFP and VGluT signals. We classified the spines that had at least 2 white overlay pixels as positive and those without overlay signals as negative. The same procedure was used to detect GluRδ2 expression. This type of analysis underestimates the percentage of PF- and CF-innervated spines, but it is suitable to compare the different groups.

To evaluate CF terminal arborization, we also measured the density of CF varicosities. The optical section images of each PC were merged to count all labeled distributed varicosities. The area of the sampled proximal dendrites and the lengths of varicosities were measured by MetaMorph imaging software (Crisel Instruments srl) to calculate the number of labeled varicosities per μm^2^ of dendritic area, major axis, and minor axis of each varicosity.

#### Golgi cell analysis

We analyzed dendritic tracts that had a diameter of approximately 0.7–0.8 µm in both the upper granular layer and molecular layer. For each Golgi cell, optical section images were merged, and we counted the spines that emerged along the dendritic tract. The spine density was expressed as the number of spines per μm of dendritic length. Colocalization of GFP and VGluT1 was calculated by MetaMorph imaging software. For each dendritic tract, we calculated the mean percentage of the entire GFP area and GFP area that overlapped with VGluT1 along the optical section images. As a negative control, we calculated the mean percentage of GFP area in the same images, represented by the Golgi axonal terminal, overlapping with the VGluT-1-labeled mossy fiber terminals in the rosette.

### Electron microscopy

GFP- or GluRδ2-expressing HEK 293 and CGC were cocultured on Aclar Fluoropolymer film (Electron Microscopy Sciences, USA). After 24 h of coculture, cells were fixed for 1 hour in 2% glutaraldehyde in PBS at room temperature, washed with cacodylate buffer, postfixed in 1% osmium tetroxide in cacodylate buffer for 1 hour on ice, and dehydrated in gradient ethanol, followed by propylene oxide. Samples were then embedded in Epon-Araldite. Ultrathin sections (80–100 nm) were cut with a diamond knife on an ultramicrotome (Leica Microsystems, Germany) and collected on Pioloform-coated single-slot grids (Electron Microscopy Sciences, USA). Sections were stained with uranyl acetate and lead citrate and examined on a JEM-1010 electron microscope (Jeol, Japan) equipped with a side-mounted CCD camera (Mega View III; Soft Imaging System, Germany).

### Ultrastructural analysis

293 cell perimeters were evaluated at 2000X magnification, using only membranes that were free of contact with GC bodies. The number of contacts between HEK cells and GC terminals was evaluated at 50,000–75,000× magnification. For each GC terminal, the length of the contact, the presence or absence of vesicles, and their distribution (spread or oriented toward the contact with the HEK cell) were considered. The vesicles were described as oriented if at least 5 were docked to the presynaptic membrane. Student's *t* test was used for statistical evaluation.

### Statistical analysis

Statistical significance was evaluated by Student's t-test, t test, or one-way ANOVA. When the interaction was significant, a post hoc test was performed for multiple comparisons. Statistical significance was assumed when p<0.05.
